# Nutritional supplementation is associated with improved wound reduction during negative pressure wound therapy in complex wounds: a retrospective cohort study

**DOI:** 10.3389/fsurg.2026.1896567

**Published:** 2026-08-24

**Authors:** Can Yahya Boztug, Ilgiz Tuzken, Attila Ulkucu, Pinar Ari, Omer Arda Cetinkaya

**Affiliations:** Department of General Surgery, Ankara University School of Medicine, Ankara, Türkiye

**Keywords:** complex wounds, malnutrition, negative pressure wound therapy, nutritional supplementation, retrospective cohort, wound area reduction, wound culture, wound healing

## Abstract

**Background:**

Negative pressure wound therapy (NPWT) is central to the management of complex wounds, but wound contraction and granulation require adequate metabolic substrate. We evaluated the association between nutritional supplementation and wound healing outcomes in patients treated with NPWT.

**Methods:**

This retrospective cohort study included 48 adults who underwent negative pressure wound therapy for complex wounds. Patients were grouped according to receipt of nutritional supplementation. The primary outcome was absence of wound reduction during follow-up.

**Results:**

Nutritional supplementation was provided to 24 patients, while 18 received no supplementation. Absence of wound reduction was less frequent with supplementation (2/24 [8.3%] vs. 8/18 [44.4%]; OR 0.114, exact 95% CI 0.011–0.750; Fisher exact *p* = 0.010), with directionally consistent findings after exploratory adjustment for body mass index and peripheral arterial disease. In the complete nutritional-risk analysis, supplemented patients had higher nutritional risk and lower visceral-protein markers; the association remained favorable after penalized adjustment for NRS-2002 score, serum albumin, baseline wound area, and wound etiology (adjusted OR 0.22, 95% CI 0.04–1.05; *p* = 0.058). Among non-amputated patients with valid serial measurements, median absolute wound-area reduction was greater in supplemented patients but narrowly missed conventional significance (15.0 [6.0–33.0] cm^2^ vs. −0.1 [−2.5–23.2] cm^2^; *p* = 0.053). Follow-up culture positivity was lower with supplementation (6/24 [25.0%] vs. 10/18 [55.6%]; *p* = 0.059). Amputation rates were similar (20.8% vs. 16.7%; *p* = 1.000), mortality was numerically higher in supplemented patients (29.2% vs. 5.6%; *p* = 0.109), and composite wound failure did not differ significantly (50.0% vs. 44.4%; *p* = 0.764).

**Conclusion:**

Nutritional supplementation was associated with a more favorable local wound trajectory during negative pressure wound therapy, despite no measurable reduction in systemic endpoints. These findings suggest that nutrition can act where negative pressure wound therapy acts first: at the wound bed.

## Introduction

Complex wounds are a persistent surgical and multidisciplinary problem, particularly among patients with diabetes mellitus, peripheral arterial disease, postoperative wound complications, soft-tissue defects, chronic inflammation, or infection. These wounds are associated with delayed recovery, repeated interventions, prolonged hospitalization, impaired quality of life, and substantial health care resource use (1, 2). Negative pressure wound therapy (NPWT) has become an established adjunctive modality for selected complex wounds by removing exudate, reducing tissue edema, improving local microperfusion, promoting granulation tissue formation, and facilitating wound contraction (3, 4).

Despite these local mechanical and biological effects, NPWT cannot compensate fully for systemic barriers to tissue repair. Wound healing requires adequate protein and caloric intake, micronutrient availability, collagen synthesis, angiogenesis, fibroblast proliferation, epithelialization, immune competence, and controlled inflammation (5, 6, 19). Patients requiring NPWT often have increased metabolic demands due to infection, chronic inflammatory activity, tissue loss, surgical stress, protein loss through wound exudate, and comorbid conditions that compromise perfusion or immune function.

Nutritional therapy is therefore biologically plausible as an adjunct to NPWT. Contemporary surgical nutrition guidance emphasizes early identification of nutritional risk and adequate nutritional support in patients with metabolic stress or impaired healing capacity (7). Reviews of chronic wound care similarly highlight the importance of protein-energy adequacy and selected nutrients in supporting immune function and tissue repair (5, 8, 9). However, clinical evidence specifically evaluating nutritional supplementation among patients undergoing NPWT remains limited, and available studies are heterogeneous with respect to wound type, nutritional formulation, nutritional risk, and outcome definitions.

Present study evaluated the association between nutritional supplementation and wound healing outcomes in patients undergoing NPWT for complex wounds. We hypothesized that nutritional supplementation would be associated with improved local wound healing dynamics, particularly lower rates of absent wound reduction, without assuming a causal effect on systemic endpoints such as mortality or amputation.

## Materials and methods

### Study design and setting

This retrospective cohort study included adult patients who underwent NPWT for complex wound management at a tertiary care center. Medical records were reviewed retrospectively. Patients were stratified into two exposure groups according to whether they received nutritional supplementation during NPWT. The study was designed and reported as an observational cohort analysis, and all causal interpretations were avoided because treatment allocation was not randomized.

### Patient selection

Adult patients treated with NPWT for chronic wounds, diabetic foot or Charcot-related wounds, peripheral arterial disease-related wounds, venous ulcers, pressure or decubitus wounds, Fournier/perineal wounds, and postoperative wound complications were eligible for inclusion. The final analytic cohort consisted of 42 patients with available clinical and wound follow-up data. Patients were excluded from the analytic dataset if clinical records were incomplete or if the wound course could not be classified for the primary endpoint.

### Exposure: nutritional supplementation

The exposure of interest was receipt of nutritional supplementation during NPWT. Supplementation was initiated as part of routine multidisciplinary care after clinical and dietetic assessment, rather than by random assignment. In practice, supplementation was considered for patients with documented or suspected nutritional risk, insufficient oral intake, high metabolic demand, extensive or exudative wounds, chronic inflammation, or impaired healing capacity.

Supplementation was predominantly oral or enteral. Wound-specific formulations containing arginine, glutamine, and beta-hydroxy-beta-methylbutyrate were commonly used, often together with diabetes-specific oral nutritional formulas in patients with diabetic wounds. A small number of patients also had parenteral nutrition documented in the product record. Omega-3-enriched supplementation was not administered according to a standardized protocol and was not reliably captured as a separate exposure. Target and recorded energy and protein prescriptions were abstracted from dietetic documentation when available.

### Nutritional and inflammatory assessment

Nutritional and inflammatory status were evaluated using body mass index, Nutritional Risk Screening 2002 score, serum total protein, prealbumin, lymphocyte count, and inflammatory markers. These variables were used to characterize nutritional risk and systemic inflammatory burden. Because the retrospective dataset did not allow complete standardization of achieved caloric and protein targets, nutritional supplementation was analyzed as a clinical exposure rather than a dose-response intervention.

### Wound evaluation and negative pressure wound therapy

NPWT was performed according to institutional wound care protocols. Wound characteristics, including wound type, anatomical localization, infection, necrosis, osteomyelitis, microbiological culture results, NPWT duration, and hospital stay were abstracted from the medical record. Wound area was abstracted from recorded wound-area measurements in cm2. Serial wound measurements were reviewed at baseline and during follow-up, including approximately week 2, week 4, and week 8 measurements when available. For continuous wound-area analyses, the latest valid follow-up wound area was compared with the baseline wound area among non-amputated patients with interpretable serial measurements. Culture findings were analyzed as documented culture positivity during the evaluated treatment/follow-up period, recognizing that culture acquisition and timing were not standardized in routine practice.

### Outcomes

The primary outcome was absence of wound reduction during follow-up. Secondary outcomes included wound area reduction over time, positive wound culture, amputation, mortality, length of hospital stay, death or amputation, and composite wound failure. Composite wound failure was defined as death, amputation, or absence of wound reduction during follow-up. This composite endpoint was considered exploratory because its components reflect different biological pathways: local tissue repair, limb perfusion and infection severity, and systemic disease burden.

### Statistical analysis

Continuous variables were summarized as median with interquartile range because of the small cohort size and non-normal distribution of several variables. Categorical variables were summarized as frequencies and percentages. Group comparisons were performed using the Mann–Whitney U test for continuous variables and Fisher exact testing for categorical variables unless otherwise specified. Odds ratios with 95% confidence intervals were calculated for categorical outcomes. Because only 10 primary outcome events were observed, multivariable analysis was intentionally limited. Exploratory logistic regression sensitivity analyses were performed for the primary endpoint using unadjusted models, selected single-covariate models, and one parsimonious two-covariate model. Penalized logistic regression was used for the complete-variable sensitivity model to reduce small-sample bias. These models were used to evaluate robustness of direction and magnitude, not to establish causality. No imputation was performed for missing data. Statistical significance was defined as a two-sided *p* value < 0.05, and all findings were interpreted in the context of the retrospective design, small sample size, and potential confounding by indication.

## Results

A total of 42 patients undergoing NPWT were included in the final analysis. Twenty-four patients received nutritional supplementation and 18 did not. Baseline clinical characteristics are summarized in Table 1. Median age was 60 years (IQR, 53.8–70.0) in the supplementation group and 67 years (IQR, 57.0–71.8) in the non-supplementation group. Body mass index was numerically lower among supplemented patients (25.1 [23.5–31.3] kg/m2 vs. 28.0 [27.4–32.1] kg/m2; *p* = 0.130). Current smoking was more frequent in the non-supplemented group (38.9% vs. 8.3%; *p* = 0.025). Diabetes mellitus, hypertension, coronary artery disease, chronic kidney disease, and peripheral arterial disease did not differ significantly between groups. Baseline total protein was lower in the supplementation group (6.22 [5.44–6.66] g/dL vs. 6.71 [6.40–6.86] g/dL; *p* = 0.025). In the complete nutritional-risk dataset, supplemented patients had higher NRS-2002 scores (4.0 [3.0–5.0] vs. 2.0 [1.0–3.0]; *p* = 0.006), lower serum albumin (2.9 [2.5–3.3] vs. 3.4 [3.1–3.8] g/dL; *p* = 0.018), and lower prealbumin (0.13 [0.10–0.18] vs. 0.19 [0.15–0.24] g/L; *p* = 0.022).

**Table 1:** Baseline clinical and laboratory characteristics.

Characteristic	Nutritional supplementation (*n* = 24)	No supplementation (*n* = 18)	*p* value
Age, years	60.0 [53.8–70.0]	67.0 [57.0–71.8]	0.373
Male sex	16/24 (66.7%)	10/18 (55.6%)	0.531
BMI, kg/m2	25.1 [23.5–31.3]	28.0 [27.4–32.1]	0.130
Current smoking	2/24 (8.3%)	7/18 (38.9%)	0.025
Diabetes mellitus	20/24 (83.3%)	16/18 (88.9%)	0.685
Hypertension	9/24 (37.5%)	11/18 (61.1%)	0.212
Coronary artery disease	5/23 (21.7%)	4/18 (22.2%)	1.000
Chronic kidney disease	5/24 (20.8%)	2/18 (11.1%)	0.679
Peripheral arterial disease	5/24 (20.8%)	6/18 (33.3%)	0.483
NRS-2002 score	4.0 [3.0–5.0]	2.0 [1.0–3.0]	0.006
Serum albumin, g/dL	2.9 [2.5–3.3]	3.4 [3.1–3.8]	0.018
Prealbumin, g/L	0.13 [0.10–0.18]	0.19 [0.15–0.24]	0.022
Total protein, g/dL	6.22 [5.44–6.66] (*n* = 20)	6.71 [6.40–6.86] (*n* = 16)	0.025
Lymphocyte count	1.46 [1.03–2.27] (*n* = 22)	1.85 [1.36–3.06] (*n* = 18)	0.232
Hemoglobin, g/dL	10.0 [8.6–11.8] (*n* = 19)	12.2 [9.6–13.7] (*n* = 17)	0.140
C-reactive protein, mg/L	60.9 [21.4–100.0] (*n* = 22)	20.6 [10.1–76.7] (*n* = 18)	0.178
White blood cell count	9.70 [7.06–10.89] (*n* = 21)	8.79 [6.77–10.90] (*n* = 17)	0.907

Values are median [IQR] or n/N (%). BMI was recalculated from recorded height and weight. Continuous variables were compared with the Mann–Whitney U test; categorical variables were compared with Fisher exact testing unless otherwise specified.

Wound-related and treatment characteristics are presented in Table 2. Wound etiology was heterogeneous. Diabetic or Charcot-related wounds were the most common wound type in both groups but were more frequent among non-supplemented patients (15/18 [83.3%]) than supplemented patients (12/24 [50.0%]). Peripheral arterial disease-related, pressure/decubitus, venous, and Fournier/perineal wounds were more frequently represented in the supplemented group. Baseline wound area among patients with valid documented area measurements was 41.5 [14.0–73.8] cm2 in supplemented patients and 20.4 [1.7–53.3] cm2 in non-supplemented patients (*p* = 0.165). In the complete wound-area dataset, baseline wound area remained numerically larger among supplemented patients (43.0 [18.5–75.0] cm2 vs. 22.5 [5.0–54.0] cm2; *p* = 0.098), and diabetic/Charcot wound etiology was more common in the non-supplemented group (50.0% vs. 83.3%; *p* = 0.054). Clinical infection, necrosis, osteomyelitis, and baseline culture positivity were not significantly different between groups.

**Table 2:** Wound-related and treatment characteristics.

Characteristic	Nutritional supplementation (*n* = 24)	No supplementation (*n* = 18)	*p* value
Diabetic/Charcot wound	12/24 (50.0%)	15/18 (83.3%)	Descriptive
Peripheral arterial disease-related wound	4/24 (16.7%)	1/18 (5.6%)	Descriptive
Pressure/decubitus wound	3/24 (12.5%)	0/18 (0.0%)	Descriptive
Venous ulcer	3/24 (12.5%)	1/18 (5.6%)	Descriptive
Fournier/perineal wound	2/24 (8.3%)	0/18 (0.0%)	Descriptive
Postoperative/surgical wound	0/24 (0.0%)	1/18 (5.6%)	Descriptive
Foot/heel location	14/24 (58.3%)	14/18 (77.8%)	Descriptive
Lower leg location	5/24 (20.8%)	3/18 (16.7%)	Descriptive
Hip/back location	3/24 (12.5%)	1/18 (5.6%)	Descriptive
Perineal/perianal location	2/24 (8.3%)	0/18 (0.0%)	Descriptive
Clinical infection documented	18/24 (75.0%)	10/18 (55.6%)	0.208
Necrosis	7/24 (29.2%)	6/18 (33.3%)	1.000
Osteomyelitis	0/24 (0.0%)	1/18 (5.6%)	0.429
Baseline culture positivity	18/24 (75.0%)	14/18 (77.8%)	1.000
Baseline wound area, cm2	41.5 [14.0–73.8] (*n* = 18)	20.4 [1.7–53.3] (*n* = 14)	0.165
Baseline wound area, cm2	43.0 [18.5–75.0] (*n* = 24)	22.5 [5.0–54.0] (*n* = 18)	0.098
Diabetic/Charcot wound vs. all other etiologies	12/24 (50.0%)	15/18 (83.3%)	0.054
NPWT duration, days	60 [30–60]	30 [30–60]	0.224

Values are median [IQR] or n/N (%). Wound-type categories were harmonized from source clinical labels. Wound etiology and location were summarized descriptively because of sparse cells across categories. NPWT indicates negative pressure wound therapy.

Nutritional supplementation details are shown in Table 3. Supplementation was predominantly oral or enteral, and most supplemented patients received a wound-specific arginine/glutamine/beta-hydroxy-beta-methylbutyrate-containing formulation. The median target energy prescription was 28.0 kcal/kg/day (IQR, 26.0–31.5), and the median target protein prescription was 1.45 g/kg/day (IQR, 1.30–1.52). The median recorded intake was 22.0 kcal/kg/day (IQR, 20.0–25.2) and 1.20 g/kg/day of protein (IQR, 0.90–1.30). The median duration of nutritional therapy was 16.0 days (IQR, 8.5–30.2). Eighteen of 24 supplemented patients (75.0%) achieved at least 80% of the prescribed energy target, and 16 of 24 (66.7%) achieved at least 80% of the prescribed protein target; the median proportion of prescribed protein delivered was 82% [72–91].

**Table 3:** Nutritional supplementation details among supplemented patients.

Variable	Value
Any nutritional supplementation	24/24 (100%)
Oral/enteral supplementation without documented parenteral support	22/24 (91.7%)
Combined enteral plus parenteral support	1/24 (4.2%)
Parenteral nutrition documented in product record	2/24 (8.3%)
Wound-specific arginine/glutamine/beta-hydroxy-beta-methylbutyrate-containing formulation	23/24 (95.8%)
Diabetes-specific oral nutritional formula	17/24 (70.8%)
High-protein oral nutritional formula	1/24 (4.2%)
Omega-3-enriched protocol	Not standardized/not reliably documented
Target energy prescription, kcal/kg/day	28.0 [26.0–31.5]
Target protein prescription, g/kg/day	1.45 [1.30–1.52]
Recorded energy intake, kcal/kg/day	22.0 [20.0–25.2]
Recorded protein intake, g/kg/day	1.20 [0.90–1.30]
Duration of nutritional therapy, days	16.0 [8.5–30.2]
Achieved at least 80% of prescribed energy target	18/24 (75.0%)
Achieved at least 80% of prescribed protein target	16/24 (66.7%)
Proportion of prescribed protein delivered	82% [72–91]

Values are median [IQR] or n/N (%). Supplementation was not protocolized and was analyzed as a pragmatic clinical exposure. Product classes were summarized from routine clinical documentation rather than a prespecified formula protocol.

**Figure 1:**
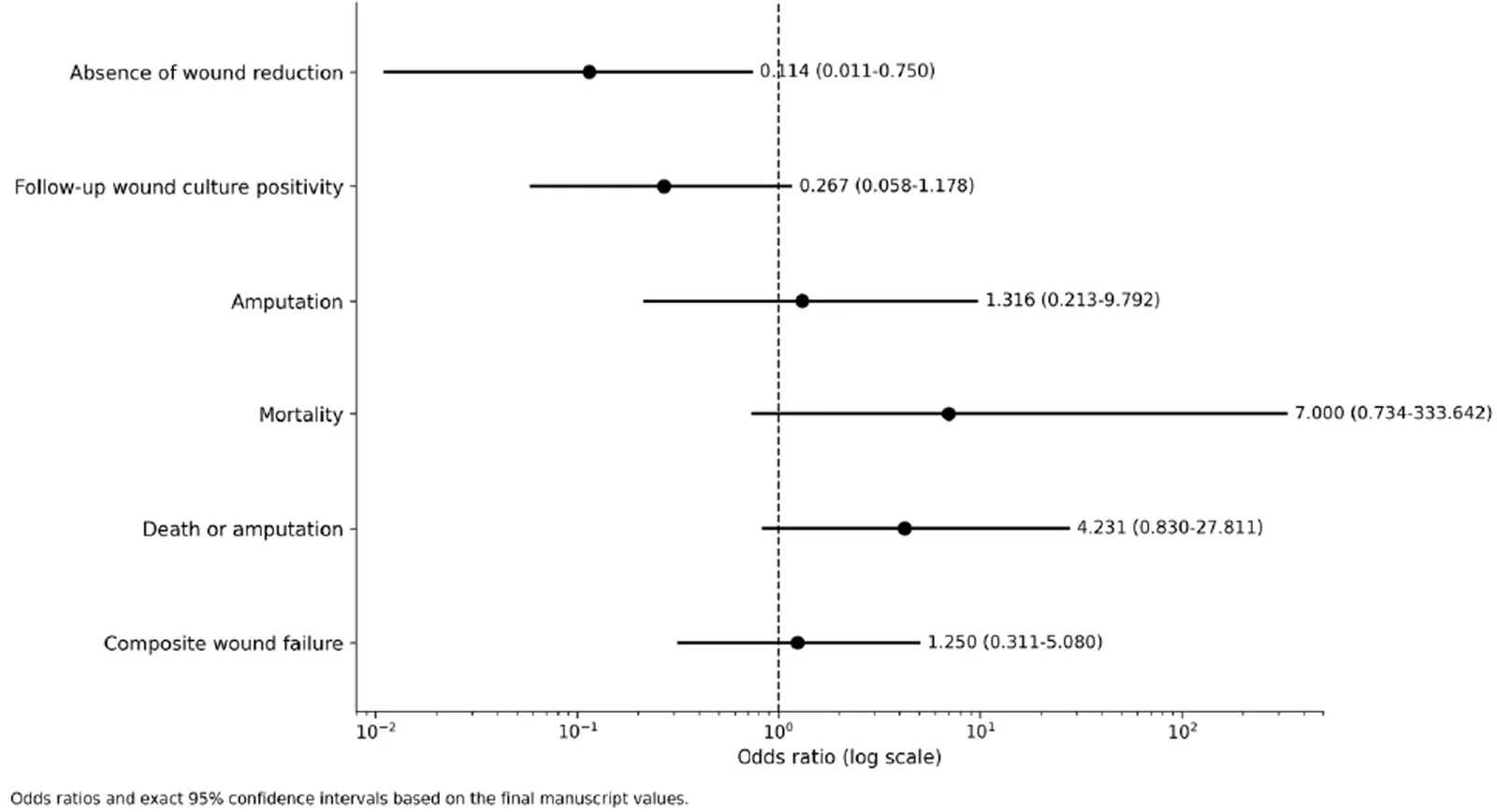
Forest plot of wound-related and clinical outcomes. Odds ratios compare the supplementation group with the no-supplementation group; values below 1 favor nutritional supplementation for the respective adverse outcome. Horizontal lines represent exact 95% confidence intervals, and the dashed vertical line indicates no effect (OR=1).

Absence of wound reduction was observed in 2 of 24 patients in the supplementation group and 8 of 18 patients in the non-supplementation group (8.3% vs. 44.4%). Nutritional supplementation was associated with lower odds of absent wound reduction (OR 0.114; exact 95% CI 0.011–0.750; Fisher exact *p* = 0.010) (Table 4; Figure 1). In exploratory logistic regression sensitivity analyses, the association remained directionally consistent after adjustment for individual baseline variables and in a parsimonious model adjusted for body mass index and peripheral arterial disease (OR 0.138; 95% CI 0.024–0.804; *p* = 0.028) (Table 5). These models were considered sensitivity analyses because only 10 primary outcome events were available. After incorporating NRS-2002 score, serum albumin, baseline wound area, and wound etiology, the association between supplementation and lower absence of wound reduction remained directionally favorable in a penalized logistic model (adjusted OR 0.22; 95% CI 0.04–1.05; *p* = 0.058). When diabetic/Charcot wound etiology was forced into the same model, the estimate was similar but less precise (adjusted OR 0.26; 95% CI 0.05–1.29; *p* = 0.095).

**Table 4:** Local wound and microbiological outcomes.

Outcome	Nutritional supplementation	No supplementation	Effect estimate/*p* value
Absence of wound reduction	2/24 (8.3%)	8/18 (44.4%)	OR 0.114; exact 95% CI 0.011–0.750; Fisher exact *p* = 0.010
Absolute wound-area reduction, cm2	15.0 [6.0–33.0] (*n* = 13)	−0.1 [−2.5 to 23.2] (*n* = 12)	*p* = 0.053
Percentage wound-area reduction	46.7% [25.0–53.2] (*n* = 13)	8.4% [−57.4 to 48.9] (*n* = 12)	*p* = 0.165
Follow-up wound culture positivity	6/24 (25.0%)	10/18 (55.6%)	OR 0.267; exact 95% CI 0.058–1.178; Fisher exact *p* = 0.059
Complete closure	2/24 (8.3%)	1/18 (5.6%)	OR 1.545; exact 95% CI 0.074–96.462; *p* = 1.000

The primary endpoint was absence of wound reduction. Continuous wound-area reduction was calculated only among non-amputated patients with valid serial area measurements, because post-amputation area does not represent biological wound contraction. Follow-up culture positivity was interpreted as culture positivity rather than a standardized clinical infection endpoint.

**Table 5:** Exploratory logistic regression sensitivity analyses for absence of wound reduction.

Model	Odds ratio for supplementation	Model-based 95% CI	*p* value
Unadjusted logistic model	0.114	0.020–0.635	0.013
Adjusted for BMI	0.126	0.022–0.715	0.019
Adjusted for age	0.106	0.017–0.648	0.015
Adjusted for peripheral arterial disease	0.120	0.021–0.679	0.016
Adjusted for necrosis	0.104	0.017–0.626	0.013
Adjusted for baseline culture positivity	0.108	0.019–0.625	0.013
Parsimonious model adjusted for BMI and peripheral arterial disease	0.138	0.024–0.804	0.028
Penalized model adjusted for NRS-2002, albumin, and baseline wound area	0.22	0.04–1.05	0.058
Penalized model adjusted for NRS-2002, albumin, baseline wound area, and diabetic/Charcot etiology	0.26	0.05–1.29	0.095

These models were exploratory because only 10 primary outcome events were observed. They were used to evaluate robustness of direction and magnitude, not to establish causality.

Among non-amputated patients with valid serial wound-area measurements, median absolute wound-area reduction was 15.0 [6.0–33.0] cm2 in the supplementation group and −0.1 [−2.5–23.2] cm2 in the non-supplementation group (*p* = 0.053). Median percentage wound-area reduction was 46.7% [25.0–53.2] and 8.4% [−57.4–48.9], respectively (*p* = 0.165). These continuous wound-area analyses were secondary and limited by missing or non-interpretable serial measurements.

Follow-up wound culture positivity was identified in 6 of 24 patients in the supplementation group and 10 of 18 patients in the non-supplementation group (25.0% vs. 55.6%; OR 0.267; exact 95% CI 0.058–1.178). This secondary finding was interpreted cautiously because Fisher exact testing yielded *p* = 0.059 and because culture acquisition and timing were based on routine clinical practice rather than a standardized protocol. Complete closure was uncommon and did not differ significantly between groups (8.3% vs. 5.6%; *p* = 1.000).

Systemic outcomes are shown in Table 6. Amputation occurred in 5 of 24 supplemented patients and 3 of 18 non-supplemented patients (20.8% vs. 16.7%; OR 1.316; exact 95% CI 0.213–9.792; *p* = 1.000). Mortality occurred in 7 of 24 supplemented patients and 1 of 18 non-supplemented patients (29.2% vs. 5.6%; OR 7.000; exact 95% CI 0.734–333.642; *p* = 0.109). Hospital stay was numerically longer among supplemented patients (60 [30–90] days vs. 32 [30–39] days; *p* = 0.079). The combined endpoint of death or amputation occurred in 11 of 24 supplemented patients and 3 of 18 non-supplemented patients (45.8% vs. 16.7%; OR 4.231; exact 95% CI 0.830–27.811; *p* = 0.057). Composite wound failure occurred in 12 of 24 supplemented patients and 8 of 18 non-supplemented patients (50.0% vs. 44.4%; OR 1.250; exact 95% CI 0.311–5.080; *p* = 0.764).

**Table 6:** Systemic and composite outcomes.

Outcome	Nutritional supplementation (*n* = 24)	No supplementation (*n* = 18)	Effect estimate/*p* value
Amputation	5/24 (20.8%)	3/18 (16.7%)	OR 1.316; exact 95% CI 0.213–9.792; *p* = 1.000
Mortality	7/24 (29.2%)	1/18 (5.6%)	OR 7.000; exact 95% CI 0.734–333.642; *p* = 0.109
Readmission	5/24 (20.8%)	2/18 (11.1%)	OR 2.105; exact 95% CI 0.289–24.536; *p* = 0.679
Hospital stay, days	60 [30–90]	32 [30–39]	*p* = 0.079
Death or amputation	11/24 (45.8%)	3/18 (16.7%)	OR 4.231; exact 95% CI 0.830–27.811; *p* = 0.057
Composite wound failure	12/24 (50.0%)	8/18 (44.4%)	OR 1.250; exact 95% CI 0.311–5.080; *p* = 0.764

Composite wound failure was defined as death, amputation, or absence of wound reduction. Because these components reflect different biological pathways, this endpoint should be interpreted as exploratory.

## Discussion

This study identifies a clinically coherent and deliberately bounded signal: among patients treated with NPWT for complex wounds, nutritional supplementation was associated with a lower frequency of absent wound reduction, whereas amputation, mortality, and composite systemic endpoints did not improve. Continuous wound-area analysis pointed in the same direction, with a larger median absolute reduction among supplemented patients, but it was confined to patients with valid serial measurements and remained underpowered. Interpretation should therefore be anchored to the prespecified categorical endpoint, not to an inflated claim of accelerated closure.

Biological rationale is strong. NPWT improves the wound-bed environment by evacuating exudate, reducing edema, stimulating granulation, improving local perfusion, and applying mechanical deformation to the wound surface (3, 4). These local effects require substrate. Fibroblast proliferation, collagen deposition, angiogenesis, epithelial migration, leukocyte function, and remodeling depend on protein-energy delivery and micronutrient availability (5, 6, 10, 11). Complex wounds are uniquely vulnerable to substrate failure because tissue loss, inflammation, bacterial burden, exudative protein loss, repeated debridement, and vascular comorbidity increase demand while reducing reserve (2, 5). In this setting, nutrition should be viewed not as a cosmetic adjunct but as part of the physiologic platform on which local wound therapy operates.

Nutritional exposure should be interpreted pragmatically. This was not a trial of a single proprietary formula or a controlled metabolic intervention. Most supplemented patients received oral or enteral support, commonly including wound-specific formulations containing arginine, glutamine, and beta-hydroxy-beta-methylbutyrate. Arginine is relevant to nitric oxide synthesis, collagen deposition, lymphocyte function, and immune modulation (12–14). Glutamine supports rapidly dividing and immune-active cells and has been associated with improved wound closure in selected trauma populations (15). Randomized and meta-analytic pressure-ulcer data also support disease-specific formulas enriched with arginine, zinc, and antioxidants for selected wound-healing endpoints (16–18). Those data cannot be mechanically transferred to NPWT-treated diabetic, ischemic, postoperative, or perineal wounds; they do, however, provide a credible biologic scaffold for the association observed in this cohort.

Delivery data strengthen the exposure definition. Most supplemented patients achieved at least 80% of prescribed energy and protein targets, supporting interpretation of the exposure as delivered nutritional therapy rather than prescription alone. This distinction is clinically important because the biology of wound repair depends less on the product label than on whether adequate amino-acid and energy substrate reached the patient during active granulation.

Baseline data are the central reason for restraint. Supplemented patients had lower baseline total protein, documented nutritional risk screening, and longer hospitalization, suggesting that clinicians preferentially prescribed supplementation to patients judged to be nutritionally vulnerable or systemically catabolic. Conversely, smoking and diabetic or Charcot-related wounds were more frequent among non-supplemented patients. These imbalances can alter wound behavior independently of nutrition. Exploratory adjusted analyses preserved the direction of the primary association, but only 10 primary events were available; such models are useful as sensitivity analyses, not as proof of an independent treatment effect.

Complete nutritional-risk comparison further supports confounding by indication rather than refuting the primary finding. Supplemented patients had higher NRS-2002 scores and lower albumin and prealbumin, a pattern consistent with the catabolic phenotype that surgical teams usually select for nutritional intervention. Albumin and prealbumin are imperfect nutritional markers in complex wounds because inflammation and capillary leak can suppress their values independent of intake; nevertheless, when interpreted alongside NRS-2002 and delivered protein-energy support, they help characterize the physiologic vulnerability of the treated group. The directionally favorable penalized estimate after adjustment for nutritional risk, visceral-protein markers, wound size, and wound etiology is consistent with the wound-nutrition literature linking substrate delivery, immune competence, collagen synthesis, and granulation to measurable local repair endpoints (5, 6, 10, 11, 16–18).

Wound biology cannot be collapsed into a single category. A diabetic foot wound, venous ulcer, pressure wound, Fournier wound, and peripheral arterial disease-related defect fail through different dominant mechanisms: off-loading failure, glycemic injury, venous hypertension, pressure, ischemia, infection, tissue loss, or a combination of these. This heterogeneity reflects real surgical wound practice and increases clinical relevance, but it reduces biologic specificity. The favorable primary endpoint across a mixed cohort is therefore noteworthy, but future studies should stratify by wound mechanism, vascular status, infection burden, and baseline nutritional risk rather than treating all NPWT wounds as metabolically equivalent.

Culture signal is supportive but not definitive. Follow-up culture positivity was less frequent among supplemented patients, which is compatible with improved host defense, reduced persistent bioburden, or a wound bed more capable of progressing through inflammation. However, culture positivity is not clinical infection, and cultures obtained in routine practice are triggered by clinical judgment rather than fixed surveillance time points. The exact confidence interval crossed unity and Fisher exact testing did not reach conventional significance. The defensible conclusion is therefore limited: culture results are directionally consistent with the local healing signal, not proof that supplementation prevents infection.

Systemic outcomes define the boundary of the paper. Supplementation was not associated with lower amputation or mortality. Mortality was numerically higher in supplemented patients, most plausibly reflecting confounding by indication and the preferential treatment of frailer, more inflamed, or more catabolic patients. This is not evidence of harm from nutrition, but it prevents any claim that short-course supplementation alters survival or limb salvage. Amputation and death in this population are often governed by ischemia, osteomyelitis, sepsis, renal disease, frailty, and global physiologic reserve - conditions that nutritional support may accompany but cannot by itself reverse.

Practical message is integration rather than isolated attribution of benefit to supplementation. Patients selected for NPWT should undergo early nutritional screening, and dietitian involvement should be embedded in the wound-care pathway. Nutritional optimization should proceed alongside debridement, source control, vascular assessment, infection management, off-loading, glycemic control, and longitudinal surgical follow-up. The role suggested by these data is support of the local wound trajectory, not replacement of definitive surgical, vascular, or infectious disease management.

This study addresses a clinically relevant NPWT population and reports both local wound outcomes and broader clinical endpoints. Primary endpoint - absence of wound reduction - is a practical outcome encountered in surgical wound care and is less dependent on complete closure than many chronic-wound endpoints. Because treatment allocation reflected real-world clinical practice and wound etiologies varied between groups, the findings should be interpreted with appropriate consideration of potential residual confounding. Future prospective studies should evaluate standardized nutritional protocols during NPWT, including objective nutritional risk screening, defined protein and caloric targets, documentation of achieved intake, and prespecified supplement composition.

## Conclusion

Nutritional supplementation was associated with lower absence of wound reduction among patients undergoing NPWT for complex wounds. Continuous wound-area data and follow-up culture results were directionally consistent with a favorable local wound trajectory, but these secondary findings were limited by incomplete serial measurements and non-standardized culture acquisition. No improvement was observed in systemic endpoints such as mortality or amputation. The association remained directionally consistent after exploratory penalized adjustment for nutritional risk, visceral-protein markers, wound size, and wound etiology, but the small number of events still precludes causal inference. These findings support systematic nutritional assessment and optimization as adjunctive components of multidisciplinary NPWT care.

## Data Availability

The raw data supporting the conclusions of this article will be made available by the authors, without undue reservation.
